# Risk of depression in patients with oral cancer: a nationwide cohort study in Taiwan

**DOI:** 10.1038/s41598-021-02996-4

**Published:** 2021-12-07

**Authors:** Ling-Yu Kung, Tsung-I Li, Chi-Hsiang Chung, Shiao-Pieng Lee, Gunng-Shinng Chen, Wu-Chien Chien, Nian-Sheng Tzeng

**Affiliations:** 1 Department of Family Dentistry and Oral Diagnosis, Tri-Service General Hospital, Taipei, Taiwan, ROC; 2 School of Dentistry, National Defense Medical Center, Taipei, Taiwan, ROC; 3 School of Public Health, National Defense Medical Center, Taipei, Taiwan, ROC; 4 Department of Medical Research, Tri-Service General Hospital, National Defense Medical Center, 7115R, No. 325, Section 2, Cheng-Gung Road, Nei-Hu District, Taipei, 11490 Taiwan, ROC; 5 Taiwanese Injury Prevention and Safety Promotion Association (TIPSPA), Taipei, Taiwan, ROC; 6 Department of Oral and Maxillofacial Surgery, Tri-Service General Hospital, National Defense Medical Center, Taipei, Taiwan, ROC; 7 Department of Orthodontics and Pediatrics Dentistry, School of Dentistry, Tri-Service General Hospital, National Defense Medical Center, Section 2, Cheng-Gung Road, Nei-Hu District, Taipei, Taiwan, ROC; 8 Graduate Institute of Life Sciences, National Defense Medical Center, Taipei, Taiwan, ROC; 9 Department of Psychiatry, School of Medicine, Tri-Service General Hospital, National Defense Medical Center, 325, Section 2, Cheng-Gung Road, Nei-Hu District, Taipei, Taiwan, ROC; 10 Student Counseling Center, National Defense Medical Center, Taipei, Taiwan, ROC

**Keywords:** Psychology, Oral cancer

## Abstract

This study investigates an association between oral cancers and the risk of developing depression. We conducted a total of 3031 patients with newly diagnosed oral cancers and 9093 age-, sex-, and index year-matched controls (1:3) from 2000 to 2013 were selected from the National Health Insurance Research Database (NHIRD) of Taiwan. After adjusting for confounding factors, multivariate Cox proportional hazards analysis was used to compare the risk of depression over a 13-year follow-up. Of the patients with oral cancer, 69 (2.28%, or 288.57 per 105 person-years) developed depression compared to 150 (1.65%, 135.64 per 105 person-years) in the control group. The Cox proportional hazards regression analysis showed that the adjustment hazard ratio (HR) for subsequent depression in patients with oral cancer diagnosed was 2.224 (95% Confidence Interval [CI] 1.641–3.013, *p* < 0.001). It is noteworthy that in the sensitivity analysis is the adjusted HR in the group with depression diagnosis was 3.392 and in the oral cancer subgroup of “Tongue” was 2.539. This study shows oral cancer was associated with a significantly increased risk for developing subsequent depression and early identification and treatment of depression in oral cancer patients is crucial.

## Introduction

Head and neck cancers are the 7th most common cancer, the 5th most common in men and the 12th most common in women worldwide^1^. Almost 50% of head and neck cancers arise in the oral cavity, there were an estimated 355,000 new cases and 177,000 deaths worldwide for oral cavity cancer in 2018^2^. Furthermore, there are approximately 4100 newly diagnosed oral cavity cancer cases in Taiwan annually, which ranks the 4th most common cancer in males and the leading cause of cancer death in males^3,4^. More than half of patients diagnosed with oral cavity cancer are in stage III or IV of the disease^4^. This requires major treatments such as radical excision, radiation therapy (RT), and surgery with concurrent chemoradiation therapy (CCRT)^5^. These treatments can cause severe physical and functional impairments that are also associated with the development of depression^6,7^. Changes in overall symptom severity, including swallowing difficulties, poor appetite, oral mucositis, pain, fatigue, and a dysmorphic appearance were all found to be significantly related to the severity of oral-cancer patients’ depression. Psychological problems require careful monitoring and special attention after treatment^8^.

Depression is a major public health problem and has an especially large impact on physical health when it appears as a comorbidity to a chronic medical condition^9^. Depression has been found to be related to cancer patient symptom severity and level of physical functioning^10,11^. Lower survival rates and poorer outcomes have been demonstrated in cancer patients with depression^12–14^. Recent evidence pointed out that depression can be seen as an independent factor for cancer survival^15^. The need for comprehensive assessments and routine symptom evaluation in management plans are emphasized because of the significant prevalence and severity of depression among new and follow-up cancer patients^16^. Overall, depression adversely affected patient quality of life and may also interfere with treatments and rehabilitation^17,18^.

Previous studies have reported an association between depression and survival in patients with head and neck (HN) cancer^19,20^. Up to 2021, only one systemic review^21^ study that link oral cavity cancer as a risk factor for depression with varied severity of symptoms after cancer treatment. Among the previous studies, Jansen et al. (2018)^22^ and Kuma et al. (2018)^23^, using validated questionnaires, found that oral cancer is associated with the risk of depression. In addition, Rana et al. (2015)^24^ have compared the risk of depression in the patients with oral squamous cell carcinoma and oral lichen planus.

We hypothesize that oral cancer is associated with an increased risk of subsequent depression. Therefore, we conducted this study to identify the association between oral cancer and depression and the potential effect modifiers, by using data from a nationwide health insurance database, the Taiwan National Health Insurance Research Database (NHIRD).

## Results

**Figure 1:**
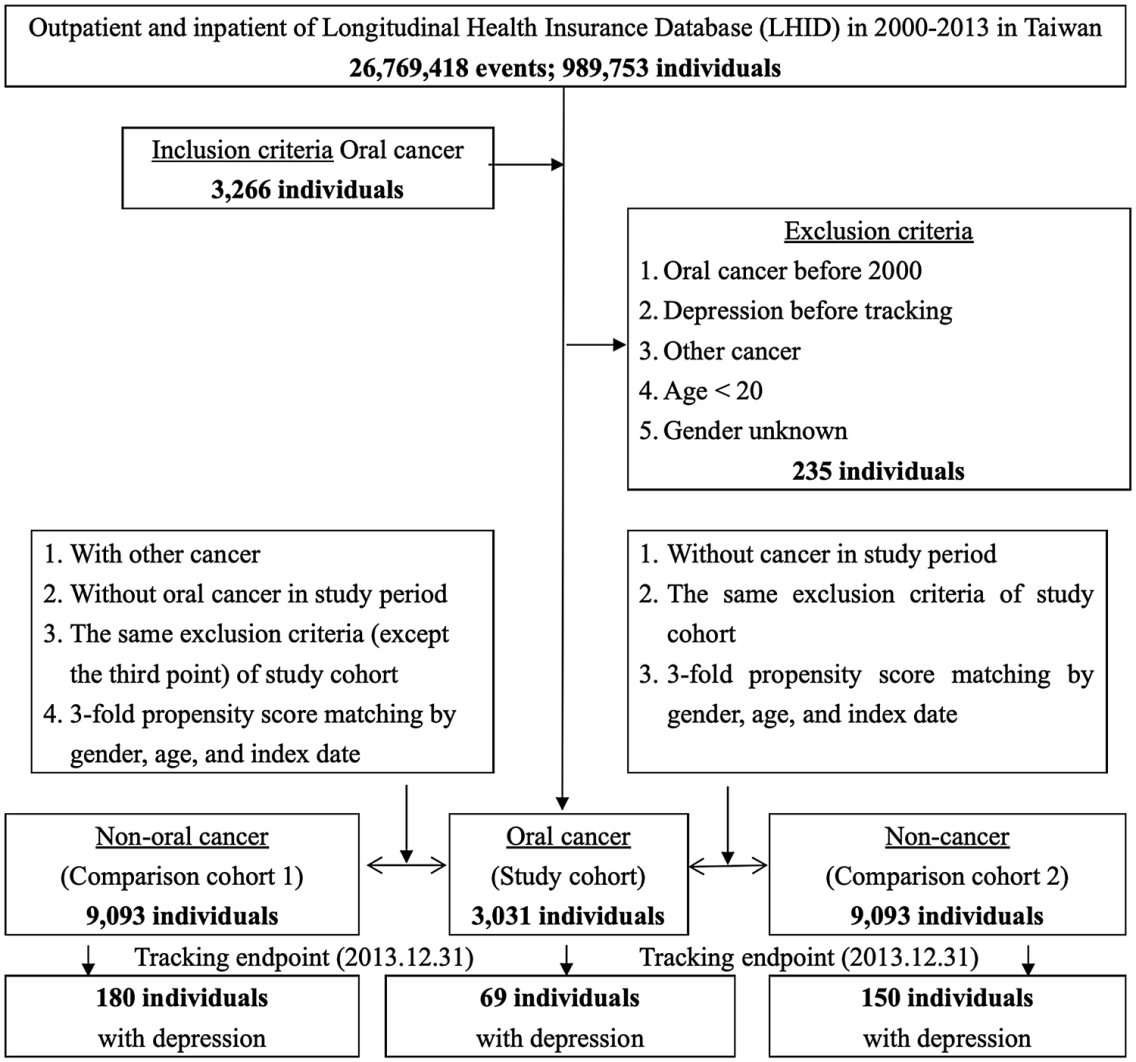
The flowchart of study sample selection from National Health Insurance Research Database in Taiwan.

Figure 1 shows the flowchart of study sample selection from National Health Insurance Research Database in Taiwan.

**Figure 2:**
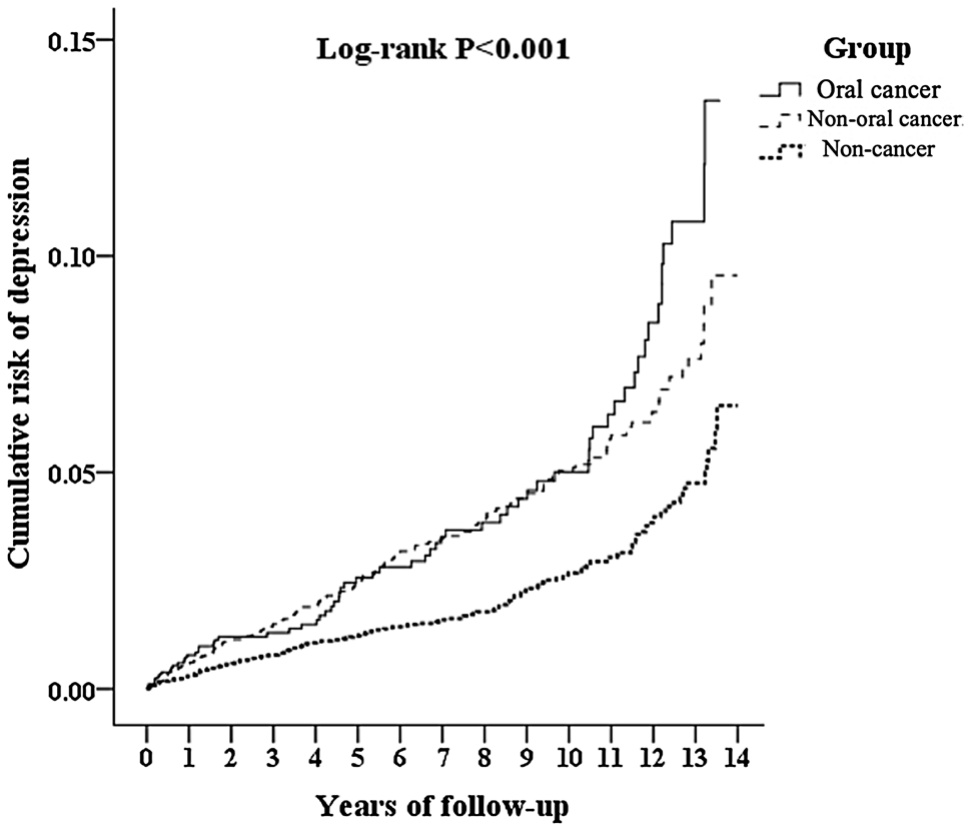
Kaplan–Meier for cumulative risk of depression among patients aged 20 and over stratified by study group with log-rank test.

Figure 2 shows the Kaplan–Meier analysis for the cumulative incidence of depression in the study and control groups. In addition, at first year of follow-up, the difference between the oral cancer group and non-cancer group became significant (log-rank test *p* < 0.001). However, the difference between oral cancer group and non-oral cancer group does not achieve significant (log-rank test *p* = 0.199).

Table 1 shows the gender, age, comorbidities, urbanization and area of residence, and income of the study subjects and controls. Most of the oral cancer patients were male in middle age (40–69) along with lower Insured premiums (NT < 18,000). Compared to the controls (non-cancer (C1)), the study subjects tended to have lower rates of alcohol abuse (0.001), IHD (*p* < 0.001), stroke (*p* < 0.001), and anxiety (*p* = 0.004).

**Table 1:** Characteristics of study in the baseline.

Variables/group	Oral cancer	Non-oral cancer	Non-cancer
	(N = 3031)	(N = 9093)	(N = 9093)
Gender male	87.96%	87.96%	87.96%
Female	12.04%	12.04%	12.04%
Age (years)	53.54 ± 12.66	53.76 ± 13.05	53.46 ± 13.31
**Age**			
20–29	1.78%	1.78%	1.78%
30–39	12.70%	12.70%	12.70%
40–49	28.67%	28.67%	28.67%
50–59	26.13%	26.13%	26.13%
60–69	19.4%	19.4%	19.4%
≧ 70	11.32%	11.32%	11.32%
**Insured premium (NT$)**			
< 18,000	92.61%	92.62%	92.58%
18,000–34,999	5.64%	5.62%	5.69%
≧ 35,000	1.75%	1.76%	1.74%
DM	5.05%	6.71%*	10.92%**
Hyperlipidemia	0.26%	1.09%**	3.52%**
HTN	3.73%	6.04%**	12.82%**
Alcohol abuse	0.10%	0.19%	0.49%**
Tobacco use	0	0	0
Stroke	1.25%	1.73%	7.32%**
COPD	1.25%	3.31%**	6.03%**
IHD	2.08%	2.23%	7.23%**
Renal disease	1.48%	2.66%**	3.48%**
Anxiety	0	0.03%	0.23%*
Sleep disorder	0.20%	0.16%	0.40%
**Location**			
Northern Taiwan	40.35%	44.59%	38.85%
Middle Taiwan	22.83%	23.18%	27.12%
Southern Taiwan	32.40%**	27.66%	26.46%
Eastern Taiwan	4.39%	4.28%	6.96%
Outlets islands Taiwan	0.03%	0.29%	0.60%
**Urbanization**			
1 (Highest) level	52.36%**	45.08%	33.65%
2	40.12%	41.27%	41.49%
3	2.38%	4.51%	7.68%
4 (Lowest)	5.15%	9.14%	17.18%
**Level of care**			
Hospital Center	76.21%**	50.61%	28.26%
Regional Hospital	15.80%	27.68%	29.65%
Local Hospital	7.98%	21.71%	42.09%
Length of days	16.27 ± 15.65**	11.08 ± 11.48	7.99 ± 9.98
Medical cost (NT$)	62,327 ± 86,318**	55,602 ± 84,346	34,104 ± 61,343

Significant values are in bold.

P: Chi-square/Fisher exact test on category variables and t-test on continue variables.

*NT$* New Taiwan Dollars, *DM* diabetes mellitus, *HTN* hypertension, *COPD* chronic obstructive pulmonary disease, *IHD* ischemic heart disease.

*The pair of subsets showed a significant difference at *p* < 0.05.

**The pair of subsets showed a significant difference at *p* < 0.001.

Compared to both controls (non-cancer (C1) and non-oral cancer(C2)), the study subjects tended to have lower rates of DM (C1 *p* < 0.001; C2 *p* = 0.001), hyperlipidemia, HTN, COPD, and renal disease (*p* < 0.001); and more lived in the highest urbanized areas, southern areas of Taiwan (*p* < 0.001), and more receive treatment in a hospital center with higher medical costs (NT$) and had longer length of days (*p* < 0.001).

Table 2 shows the results of Cox regression analysis of the factors associated with the risk of developing depression. In oral cancer vs. non-cancer group, the crude HR was 2.181 (95% CI = 1.637–2.906, *p* < 0.001). After adjusting for age, gender, comorbidities, geographical area of residence, urbanization level of residence, and monthly income, the adjusted HR was 2.224 (95% CI = 1.649–3.028, *p* < 0.001).

**Table 2:** Factors of depression by using Cox regression.

Variables	Oral cancer versus non-cancer	
	Crude HR (95% CI)	Adjusted HR (95% CI)
Oral cancer (vs Non-cancer)	2.181 (1.637–2.906)**	2.224 (1.641–3.013)**
Male (vs Female)	0.688 (0.481–0.983)*	0.687 (0.474–0.993)*
**Age (vs 20**–**29 years old)**		
30–39	1.710 (0.647–4.520)	1.648 (0.618–4.393)
40–49	1.171 (0.461–2.974)	1.205 (0.469–3.095)
50–59	0.841 (0.335–2.107)	0.870 (0.341–2.215)
60–69	0.565 (0.222–1.438)	0.589 (0.228–1.523)
≧ 70	0.681 (0.273–1.697)	0.679 (0.267–1.727)
**Insured premium (vs < 18,000 NT$)**		
18,000–34,999	0.921 (0.295–2.879)	0.971 (0.309–3.049)
DM (vs without)	0.648 (0.438–0.958)*	0.79 (0.525–1.188)
Hyperlipidemia (vs without)	0.435 (0.162–1.169)	0.428 (0.155–1.180)
Hypertension (vs without)	0.750 (0.538–1.045)	1.017 (0.709–1.457)
Alcohol abuse (vs without)	**8.012 (4.109**–15.625)**	**10.63 (5.32**–21.26)**
Stroke (vs without)	1.238 (0.833–1.841)	1.567 (1.034–2.375)*
COPD (vs without)	0.943 (0.575–1.548)	1.003 (0.597–1.686)
IHD (vs without)	0.757 (0.473–1.213)	1.108 (0.680–1.808)
Renal disease (vs without)	0.413 (0.183–0.930)*	0.519 (0.228–1.180)
Anxiety (vs without)	5.180 (1.657–16.193)*	**5.978 (1.861**–19.20)**
Sleep disorder (vs without)	**4.209 (2.077**–8.530)**	**3.109 (1.497**–6.457)**
**Location (vs Northern Taiwan)**		
Middle Taiwan	0.981 (0.698–1.380)	Had collinearity with urbanization level
Southern Taiwan	0.909 (0.635–1.30)	
Eastern Taiwan	2.048 (1.365–3.071)*	
Outlets islands	1.097 (0.153–7.893)	
**Urbanization level (vs level 4, lowest)**		
1 (The highest)	0.800 (0.553–1.159)	0.859 (0.564–1.307)
2	0.790 (0.560–1.113)	0.889 (0.617–1.281)
3	0.562 (0.293–1.080)	0.590 (0.306–1.137)
**Level of care (vs Local hospital)**		
Hospital center	0.683 (0.486–0.961)*	0.573 (0.384–0.855)*
Regional hospital	0.684 (0.495–0.945)*	0.697 (0.500–0.973)*
Length of days	**1.021 (1.013**–1.029)**	**1.014 (1.006**–1.022)**
Medical cost (NT$)	1.000 (1.000–1.001)*	Had collinearity with length of days

*HR* hazard ratio, *CI* confidence interval, *Adjusted HR* adjusted variables listed in the Table 1.

*NT$* New Taiwan Dollars, *DM* diabetes mellitus, *HTN* hypertension, *COPD* chronic obstructive pulmonary disease, *IHD* ischemic heart disease.

*The pair of subsets showed a significant difference at *p* < 0.05.

**The pair of subsets showed a significant difference at *p* < 0.001.

In oral cancer vs. non-cancer group, the adjusted HR’s for the patients with alcohol abuse, anxiety, and sleep disorder for developing depression were 10.631 (*p* < 0.001), 5.978 (*p* < 0.0013), and 3.109 (*p* < 0.001), respectively. The patients with DM and renal disease tended to have a lower risk for developing depression before adjustment; however, this became insignificant after adjustment.

The male patients adjusted HR was 0.687 (*p* = 0.047) and received treatment in hospital centers (0.573 (*p* = 0.001)) and regional hospitals (0.697 (*p* = 0.025)) tended to have a lower risk for developing depression than those who visited a local hospital. An increase in stay by 1 day also increases the risks for developing depression by 1.9% (*p* < 0.001).

Table S2 illustrated that the depression, with the validation by the suicide (ICD-9-CM codes: E950-E959) and the usage of the antidepressants, which revealed that oral cancer is associated with the depression in the subgroups either with or without suicides or the usage of antidepressants.

Table S3 depecits the different models on the matching between the oral cancer group and the controls.The adjusted SHR’s would be closer to the crude SHR’s while the covariates were more extensively matched.

Table 3 shows the results of oral cancer subgroup by using Cox regression. The adjusted HR’s for developing depression from areas such as and indicated: lips 2.379 (*p* = 0.001), tongue 2.539 (*p* < 0.001), gums 2.163 (*p* = 0.020), floor of mouth 2.791 (*p* = 0.031), Cheek mucosa 1.872 (*p* = 0.029), and Others 2.326 (*p* < 0.001).

**Table 3:** Factors of depression stratified by oral cancer subgroup by using Cox regression.

Subgroup	Oral cancer	Depression	Oral cancer vs. non-oral cancer			Oral cancer vs. non-cancer		
	n	n	Adjusted HR	95% CI	*p*	Adjusted HR	95% CI	*p*
Total	3031	69	1.112	0.834–1.482	0.424	2.224	1.641–3.013	< 0.001
Lip	231	8	1.276	0.617–2.638	0.486	2.379	1.148–4.930	0.001
Tongue	1031	26	1.368	0.900–2.080	0.127	2.539	1.645–3.920	< 0.001
Major salivary glands	35	0	0.000	–	0.944	0.000	–	0.942
Gum	194	5	1.086	0.443–2.662	0.842	2.163	1.083–5.339	0.020
Floor of mouth	118	3	1.328	0.420–4.205	0.599	2.791	1.010–8.949	0.031
Cheek mucosa	686	13	1.291	0.722–2.312	0.380	1.872	1.036–3.380	0.029
Others	736	14	0.819	0.471–1.423	0.462	2.326	1.323–4.091	< 0.001

*HR* hazard ratio, *CI* confidence interval, *Adjusted HR* adjusted variables listed in Table 2.

Table 4 shows the results of sensitivity analysis for tracking interval, in oral cancer vs. non-cancer group, interval group < 1 year 3.392 (*p* < 0.001), ≧1 year, < 3 years 2.604 (*p* < 0.001), ≧3 year, < 5 years 1.498 (*p* = 0.001), and ≧5 year 1.364 (*p* = 0.013) were associated with a higher risk developing depression and hazard ratio show decline tendency as follow-up time was longer.

**Table 4:** Sensitivity analysis for factors of depression by using Cox regression.

Tracking interval	Oral cancer vs. non-oral cancer			Oral cancer vs. non-cancer		
	Adjusted HR	95% CI	*p*	Adjusted HR	95% CI	*p*
Overall	1.112	0.834–1.482	0.424	2.224	1.641–3.013	< 0.001
< 1 year	1.407	0.897–1.678	0.306	3.392	2.243–6.665	< 0.001
≧ 1 year, < 3 years	1.295	0.855–1.592	0.398	2.604	1.725–4.076	< 0.001
≧ 3 years, < 5 years	1.112	0.831–1.466	0.433	1.498	1.127–2.791	0.001
≧ 5 years	1.098	0.792–1.403	0.501	1.364	1.035–2.005	0.013

*HR* hazard ratio, *CI* confidence interval, *Adjusted HR* adjusted variables listed in Table 2.

## Discussion

This study showed that patients with a diagnosis of oral cancer has nearly 2.2-fold increased risk of developing depression. Kaplan–Meier analysis revealed that the study subjects had a significantly lower 13-year depression-free survival rate than the controls. In addition, it took 1 year to achieve a significantly adjusted HR, and therefore, the duration of 13 years appears to be a reasonable period to follow patients with newly diagnosed oral cancer. To the best of our knowledge, this is the first population-based study on the topic of the increased risk of depression in the patients with oral cancer.

A study done in Hong Kong concluded that approximately 8% of the oral cancer study participants met the clinical cut-off for depression^25^. Another previous study on Taiwan focused on early post-surgery stage patients with oral cancer and showed that 18.2% met the definition of depression^26^. A study about the Taiwan NHIRD further indicated that newly diagnosed oral cavity cancer patients age >  = 50 had a significantly lower 5-year depression event-free survival rate with 2.53-fold hazard ratios higher than controls^27^.

Our study results show a 2.224-fold greater risk than controls that had a 2.28% oral cancer diagnosed with depression. Further, our study indicated a greater number of patients, broader age groups, and a longer follow-up period. More detailed data about each subgroup according to ICD9 area and sensitivity analysis was collected and provided. A low prevalence of depression was reported in this study, which might be rooted in the cultural context. Taiwan male adults tend to demonstrate a kind of stoicism that may be reflected in a much lower percentage in help-seeking behaviors^28^.

In this study, a patient diagnosed with oral cancer in the lip, tongue, and other cancer subgroups show a higher significant hazard ratio than others (Table 3). The other cancer subgroup were mostly composed of hard palate, soft palate, and retromolar areas. Oral cancer treatment for the lip subgroup may include surgery that involved removal of these facial features. An altered facial appearance and trouble speaking can lead to social isolation and psychological distress^29,30^.

The tongue and soft palate are the most important organs in the oral cavity and oropharynx for speech and swallowing^31^. Patients who underwent 3/4 or total anterior glossectomy had poorer functional outcomes than those who underwent either 1/4 or 1/2 glossectomy^32^. A previous study showed that depression and swallowing function were highly correlated^33^. Patients with tumors of the tongue had worse functional dysphagia than those with cancers in other areas of the oral cavity^34^. Dysphagia contribute to malnutrition, dehydration, weight loss, reduced functional abilities, and fear of eating and drinking may also lead to depression and reduced quality of life^35^. Early identification and management of dysphagia can improve treatment outcomes and reduces the development of depression^31^.

The direction of the relationship among depression, swallowing function, and quality of life for HNC patient remains unclear^33^. A recent study showed that dysfunction in salivation, problems with eating, and problems with social contact were major risk factors for the development of depression^36^.

Oral cancer represent a psychosocial challenge for patients, their families, and the diagnosing provider^29^.In Taiwan, the majority of oral cavity cancer patients belonged to a lower economic group and had a long history of smoking, chewing betel nut, and drinking alcohol^37^, which were also correlated with depression^38–40^. A synergistic effect on depression might develop from the sudden cessation of the afore-mentioned substance used after receiving the cancer diagnoses^26^.

Our study indicated that most oral cancer patients come from a lower annual income backgrounds, which may contribute to increasing the hazards for subsequent depression. A previous study showed a large percentage of patients who had received oral cancer treatment indicated a significant financial impact (treatment costs, work absences, medication prices, and other miscellaneous expenses), especially for patients with limited financial resources^41^. Financial struggle causes significant stress in patients undergoing oral cancer treatment^42^. Oral cancer survivors do not always return to work after rehabilitation^43^. Patients may encounter several physical challenges that may influence patient employability and an ability to maintain or continue employment^44^.

Oral cancer surgery often causes significant loss of function, and further radiotherapy and chemotherapy caused side-effects such as nausea, vomiting, mucositis, pharyngitis-related dysphagia, masticatory disorders, and possible loss of complete physiological functions^45,46^. During treatment, a higher consumption of painkillers, especially those with narcotic analgesics, has been reported to have a higher risk of depression^47,48^. Therefore, both oral cancer and its treatment disrupts core aspects of daily life, which may further damage a patient’s self-esteem and encourage social isolation^49^.

According to another study, there is a high incidence of symptoms of depression in HNCA patients in the first 6 months to 1 year following definitive therapy^33,36^, which is similar to our study result especially in < 1 year group, i.e. they showed a significant 3.392 hazard ratio (Table 4). It remains not clear why depression develops so early in the present and other studies. One possible reason is that the oral cancer might be related to the physical discomfort and psychosocial distress, such as dysphagia, from the facial disfigurement due to the cancer itself, since more than half of the oral cancers occur on the tongues, cheeks and lips in the present study (as shown in Table 3), that are related eating, swallowing, and body figures.

Lazure et al. found that HNC patients with depression had significantly greater mortality and disease recurrence compared to those who had not developed depression^50^. Shinn et al. found that patients with oropharynx cancer, self-reported depression was associated with a 3.6 greater hazard of death and 3.8 greater hazard of disease recurrence^12^. Further, Kam et al. found that individuals with HNC had a 3 times greater incidence of suicide compared with the general population in the United States^51^. Reike et al. conducted a study that included 3466 HNC patients with depression and showed a 1.49 higher hazard ratio died of cancer^20^. Oral cancer patients with mental illness conferred a 1.58-times risk of mortality were less likely to undergo surgery with or without adjuvant therapy and had a poor prognosis when compared with those without mental illness^26^. Previous research has also shown that depression may compromise the immune system and may affect natural killer cells important to apoptosis, which leaves individuals at greater risk for cancer development^52^. It is noteworthy, Kim et al. found that HNC patients with pre-treatment depression had a decreased 3-year survival rate when compared to their non-depressed counterparts^53^.

The prognostic impact of comorbidity on overall survival is recognized and easily explained, but on disease-specific survival it is less well understood^54^. A previous study showed lower survival rates in early stage oral cancer patients with comorbidity conditions that were due to less aggressive cancer treatments offered to this group of patients^55^. Another population-based study from Denmark concluded that comorbidity had a negative prognostic impact on overall survival rates in HNC patients, but cancer-specific death was not significantly affected by comorbidity, suggesting that patients die from or of their comorbidities rather than the cancer^56^.

Table 2 showed that oral cancer patients with sleep disorder, anxiety, and alcohol abuse showed significant hazard ratio subsequent to depression when analyzing a study population, which was correlated to previous studies^57–59^. Meanwhile, the study group showed fewer associations with other comorbidities, which might imply that oral cancer and subsequent depression should have shown a higher specificity.

## Methods

### Data sources

In this study, we used data from the NHIRD to investigate the relationship among a matched control group (non-cancer), an oral cancer group, a non-oral cancer group, and depression over a 13-year period from the Longitudinal Health Insurance Database (LHID) in Taiwan (2000–2013). The National Health Insurance (NHI) Program was launched in Taiwan in 1995, and as of June 2009 it included contracts with 97% of the medical providers in Taiwan with approximately 23 million beneficiaries, or more than 99% of the entire population in Taiwan^60^. The details of this program have been well documented in the literature^61–64^.

The NHIRD uses the International Classification of Diseases, 9th Revision, Clinical Modification (ICD-9-CM) codes to record diagnoses^65^. All diagnoses of depression were made by board-certified psychiatrists and oral cancers were confirmed by oral surgeons or otolaryngologists, and other cancers were confirmed by medical experts who specialized in oncology. The Catastrophic Illness Certificates (CIC) status was used to verify the diagnosis of oral or other cancers. The NHI Administration randomly reviews the records of 1 in 100 ambulatory care visits and 1 in 20 in-patient claims to verify the accuracy of diagnoses^66^. Several studies have demonstrated the accuracy and validity of diagnoses in the NHIRD^67–69^.

### Study design and sampled participants

This study was a retrospective matched-cohort design. Patients with newly diagnosed oral cancer were selected from 1 January 2000 to 31 December 2013 according to ICD-9-CM codes at the sites of lips, tongue, major salivary glands, gums, floor of mouth, cheek mucosa, and other sites in the oral cavity (listed in Table S1). The patients with ages younger than 20 years, any previous diagnosis of cancer, and depression before year of 2000 were excluded. The oral cancer group was matched (1:3) with the two control groups, cancers other than the oral cancer group and the non-cancer group. A total of 989,735 enrolled patients with the 3031 participants with oral cancer and age-, sex-, and index year-matched control groups as 9093 cancer other than oral cancer group and 9093 non-cancer group, respectively (Fig. 1).

The covariates included gender, age groups of the 10-year interval as 18–29, 30–39, 40–49, 50–59, 60–69, and ≥ 70 years, geographical area of residence (north, center, south, and east Taiwan), urbanization level of residence (level 1–4), and monthly income (in New Taiwan Dollars (NTD): < 18,000, 18,000–34,999, and ≥ 35,000). The urbanization level of residence was defined according to the population and various indicators of the level of development. Level 1 was defined as a population > 1,250,000, and a specific designation as political, economic, cultural, and metropolitan development. Level 2 was defined as a population between 500,000 and 1249,999, and as playing an important role in the political system, economy, and culture. Urbanization levels 3 and 4 were defined as a population between 149,999 and 499,999, and < 149,999, respectively^70^.

Baseline comorbidities included diabetes mellitus (DM), hyperlipidemia, hypertension, alcohol abuse, tobacco use, stroke, chronic obstructive pulmonary disease, ischemic heart disease, renal disease, anxiety, and sleep disorders (Table S1).

### Outcome measures

All study participants were followed from the index date until the onset of depression (ICD-9-CM codes: 296.2x, 296.3x, 300.4, and 311), withdrawal from the NHI program, or through to the end of 2013^71^.

### Statistical analysis

All analyses were performed using SPSS software version 22 (SPSS Inc., Chicago, Illinois, USA). The student’s t-tests and χ^2^ were used to evaluate the distributions of categorical and continuous variables, respectively. Multivariate Cox proportional hazards regression analysis was used to determine the risk of depression, and the results were present as hazard ratio (HR) with 95% confidence interval (CI). The difference in the risk of depression between the study (oral cancer) and control groups (non-oral cancer and non-cancer) was estimated using the Kaplan–Meier method with the log-rank test.

### Strengths

First, we conducted this population-based study, by LHID retrieved from the NHIRD, a large sample size during the duration of 13 years. A long-term observation allowed for more credibility in this study. Second, one recent study has validated the accuracy of the diagnostic code for major depressive disorder in the NHIRD and supporting the utilization of psychiatric diagnoses in claims databases^72^. Third, the validation of diagnostic codes of cancers, in the NHIRD, has been confirmed, in accordance with the Taiwan National Cancer Registry (NCR) and the Catastrophic Illness Certificates (CIC) status for oral^73^ or a wide range of most of the cancers^74^. The accuracy of the diagnostic codes for the depression or oral cancer could provide a relative reliable association between oral cancer and depression. Fourth, the validation by suicide and antidepressant usage and sensitivity analysis also revealed the association between oral cancer and depression. These findings support evidence of the association between oral cancer and the risk of depression.

### Limitation

There were several limitations to this study: First, since this is a study in a Taiwanese population, it would be limited in the generalization of the results to other countries. Second, in Taiwan, some specialists other than board-certified psychiatrists, such as neurologists or family medicine physicians, might prescribe antidepressants with the diagnosis of depressive disorder. In addition, depression is significantly underdiagnosed via ICD codes and that survey-based screening data is necessary for full screening of depression risk. Third, even though patients with the diagnosis of depression and oral cancer could be identified using the insurance claims data, the data on the severity, stage, and impact on their caregivers and socio-economic status were not available. Fourth, tobacco cessation was not included in the NHIRD, therefore, it was not matched it in this study. In addition, details regarding the cancer therapy and progression were not fully available in the NHIRD. More research may be necessary to clarify the long-term risk of developing depression in patients with oral cancer.

### Ethical approval

This study was conducted in accordance with the Code of Ethics of the World Medical Association (Declaration of Helsinki). The Institutional Review Board of Tri-Service General Hospital approved this study and exempted the need for individual written informed consent (TSGHIRB No: B-109-40).

### Informed consent

The Institutional Review Board agreed that informed consent could be waived because these databases are encrypted databases.

## Conclusion

In our study, oral cancer diagnoses were associated with a significant increased risk for the subsequent development of depression. This study serves as a reminder for dentists and physicians that early identification and treatment of depression in oral cancer patients is crucial. Future studies are needed for better treatment strategies for the oral cancer patients to help with and stave off the development of depression.

## Supplementary Information

Supplementary Table S1.

Supplementary Table S2.

Supplementary Table S3.

## Data Availability

The dataset is deposited in the website of Taiwan’s National Health Research Database (https://nhird.nhri.org.tw/en/).
