# Supplementary material for: Risk of depression in patients with oral cancer: a nationwide cohort study in Taiwan
Source: Sci Rep. 2021 Dec 7;11:23524. doi: 10.1038/s41598-021-02996-4 (PMC8651796; doi:10.1038/s41598-021-02996-4)
Supplement: Supplementary file 1 — Supplementary Table S1. [file 41598_2021_2996_MOESM1_ESM.docx]

| **Table S1. ICD-9-CM List** | |
| --- | --- |
| **Oral cancer** | **ICD-9-CM** |
| Lip | 140 |
| Tongue | 141 |
| Major salivary glands | 142 |
| Gum | 143 |
| Floor of mouth | 144 |
| Cheek mucosa | 145.0 |
| Others | 145, excluding 145.0 |
| **Non-oral cancer** | 146-239 |
| **Depression** | 296.2-296.3, 300.4, 311 |
| **Comorbidity** | **ICD-9-CM** |
| Diabetes mellitus (DM) | 250 |
| Hyperlipidemia | 272 |
| Hypertension (HTN) | 401-405 |
| Alcohol abuse | 303, 305.0, V11.3 |
| Tobacco use | 305.1 |
| Stroke | 430-438 |
| Chronic obstructive pulmonary disease (COPD) | 490-496 |
| Ischemic heart disease (IHD) | 410-414 |
| Renal disease | 580-589 |
| Anxiety | 300.00 |
| Sleep disorder | 307.4, 780.5 |
