# Supplementary material for: Risk of depression in patients with oral cancer: a nationwide cohort study in Taiwan
Source: Sci Rep. 2021 Dec 7;11:23524. doi: 10.1038/s41598-021-02996-4 (PMC8651796; doi:10.1038/s41598-021-02996-4)
Supplement: Supplementary file 2 — Supplementary Table S2. [file 41598_2021_2996_MOESM2_ESM.docx]

| **Table S2. Factors of depression subgroup by using Cox regression** | | | | | | | | | | | |
| --- | --- | --- | --- | --- | --- | --- | --- | --- | --- | --- | --- |
| **Depression subgroup** | **Events, n** | | | **Oral cancer *vs.* non-oral cancer** *(Reference)* | | | | **Oral cancer *vs.* Non-cancer** *(Reference)* | | | |
|  | **Oral cancer** | **Non-oral cancer** | **Non-cancer** | **Adjusted HR** | **95% CI** | **95% CI** | ***P*** | **Adjusted HR** | **95% CI** | **95% CI** | ***P*** |
| **Overall** | 69 | 180 | 150 | 1.112 | 0.834 | 1.482 | 0.424 | 2.224 | 1.641 | 3.013 | <0.001 |
| Without antidepressant, without suicide | 2 | 6 | 4 | 1.084 | 0.795 | 1.401 | 0.406 | 2.183 | 1.602 | 2.986 | <0.001 |
| Without antidepressant, with suicide | 11 | 25 | 19 | 1.267 | 0.843 | 1.533 | 0.497 | 2.310 | 1.711 | 3.184 | <0.001 |
| With antidepressan, without suicide | 47 | 136 | 117 | 1.433 | 0.925 | 1.572 | 0.534 | 2.562 | 1.752 | 3.245 | <0.001 |
| With antidepressant, with suicide | 9 | 13 | 10 | 1.095 | 0.811 | 1.466 | 0.418 | 2.206 | 1.635 | 3.002 | <0.001 |
| Without antidepressant | 13 | 31 | 23 | 1.173 | 0.820 | 1.468 | 0.449 | 2.246 | 1.655 | 3.084 | <0.001 |
| With antidepressantt | 56 | 149 | 127 | 1.254 | 0.867 | 1.519 | 0.472 | 2.433 | 1.731 | 3.215 | <0.001 |
| Without suicide | 49 | 142 | 121 | 1.246 | 0.859 | 1.483 | 0.467 | 2.378 | 1.693 | 3.121 | <0.001 |
| With suicide | 20 | 38 | 29 | 1.177 | 0.828 | 1.500 | 0.455 | 2.226 | 1.645 | 3.043 | <0.001 |
| **HR= hazard ratio, CI = confidence interval, Adjusted HR: Adjusted variables listed in Table 2.** | | | | | | | | | | | |
