# Supplementary material for: Risk of depression in patients with oral cancer: a nationwide cohort study in Taiwan
Source: Sci Rep. 2021 Dec 7;11:23524. doi: 10.1038/s41598-021-02996-4 (PMC8651796; doi:10.1038/s41598-021-02996-4)
Supplement: Supplementary file 3 — Supplementary Table S3. [file 41598_2021_2996_MOESM3_ESM.docx]

| **Table S3. Factors of depression by using Cox regression in different model** | | | | | | | | | |
| --- | --- | --- | --- | --- | --- | --- | --- | --- | --- |
| **Model** | **Group** | **Crude HR** | **95% CI** | **95% CI** | ***P*** | **Adjusted sHR** | **95% CI** | **95% CI** | ***P*** |
| **Model 0** | **Oral cancer** | 1.417 | 1.125 | 1.782 | <0.001 | 1.382 | 1.044 | 1.707 | 0.005 |
|  | **Without cancer** | Reference |  |  |  | Reference |  |  |  |
| **Model 1** | **Oral cancer** | 2.181 | 1.637 | 2.906 | <0.001 | 2.224 | 1.641 | 3.013 | <0.001 |
|  | **Without cancer** | Reference |  |  |  | Reference |  |  |  |
| **Model 2** | **Oral cancer** | 2.220 | 1.678 | 2.915 | <0.001 | 2.231 | 1.682 | 3.024 | <0.001 |
|  | **Without cancer** | Reference |  |  |  | Reference |  |  |  |
| **Model 3** | **Oral cancer** | 2.233 | 1.707 | 3.098 | <0.001 | 2.235 | 1.707 | 3.099 | <0.001 |
|  | **Without cancer** | Reference |  |  |  | Reference |  |  |  |
| **Model 0: unmatched** | | | | | | | | | |
| **Model 1: matched by sex, age, and index date** | | | | | | | | | |
| **Model 2: matched by sex, age, index date, and comorbidities** | | | | | | | | | |
| **Model 2: matched by sex, age, index date, comorbidities, insured premium, location, urbanization level, and level of medical care** | | | | | | | | | |
| **HR = Hazard ratio; Adjusted HR: Adjusted variables listed in Table 1; CI = confidence interval** | | | | | | | | | |
